# The Physical Diagnosis of Brain Disease

**Published:** 1871-10

**Authors:** 


*The Physical Diagnosis of Brain Disease.* By Reuben A. Vance, M.D.

In this reprint Dr. Vance advocates the use of four different instruments with which the physician may diagnose the physical signs of brain affection, and concludes as follows:

1. The thermometer indicates local variations of temperature. In some cases of nervous disorder, Dr. Brown-Sequard says that the difference between the two sides may exceed 12°.

2. The dynamometer registers the comarative strength of the two sides, and in cases of disease determines the side of the brain in which it exists with the greatest intensity.

3. In like manner, the æsthesiometer indicates the comparative sensibility of the two lateral halves of the body, and affords like information as to the site of the cerebral disease.

4. The ophthalmoscope enables us to demonstrate the condition of the cerebral circulation, and thus to discover the immediate cause of the brain symptoms. In the vast majority of cases, this will be a state of hyperæmia. In some, however, anæmia will be the cause. In certain cases, local extravasations of blood can be seen in the retinal structures, together with very intense congestion. Should organic disease be present, it, in the majority of instances, will be indicated by structural changes in the optic disc of the side on which it exists.

